# Physiological lipid composition is vital for homotypic ER membrane fusion mediated by the dynamin-related GTPase Sey1p

**DOI:** 10.1038/srep20407

**Published:** 2016-02-03

**Authors:** Shintaro Sugiura, Joji Mima

**Affiliations:** 1 Institute for Protein Research, Osaka University, Suita, Osaka, Japan

## Abstract

Homotypic fusion of the endoplasmic reticulum (ER) is required for generating and maintaining the characteristic reticular ER membrane structures. This organelle membrane fusion process depends on the ER-bound dynamin-related GTPases, such as atlastins in animals and Sey1p in yeast. Here, to investigate whether specific lipid molecules facilitate GTPase-dependent ER membrane fusion directly, we comprehensively evaluated membrane docking and lipid mixing of reconstituted proteoliposomes bearing purified Sey1p and a set of ER-mimicking lipids, including phosphatidylcholine, phosphatidylethanolamine, phosphatidylinositol, phosphatidylserine, phosphatidic acid, and ergosterol. Remarkably, we revealed that each specific lipid species contributed little to membrane docking mediated by Sey1p. Nevertheless, Sey1p-dependent lipid mixing was strongly reduced by omitting three major acidic lipids from the ER-mimicking set and, moreover, was entirely abolished by omitting either phosphatidylethanolamine or ergosterol. Our reconstitution studies thus established that physiological lipid composition is vital for lipid bilayer rearrangements in GTPase-mediated homotypic ER membrane fusion.

Intracellular organelles in eukaryotic endomembrane systems have a variety of sizes and shapes, and organelle membrane morphology is essentially linked to diverse cellular functions^1^,^2^. In particular, of these organelles, the endoplasmic reticulum (ER) has a characteristic reticular membrane structure with three-way junctions of tubular membranes^3^. Miscellaneous proteins involved in ER reticular morphology, such as reticulons, DP1/Yop1p-family proteins, dynamin-related atlastin GTPases, Lunapark-family proteins, and ER-localized soluble *N*-ethylmaleimide-sensitive factor attachment protein receptors (SNAREs)^4^,^5^,^6^,^7^,^8^,^9^, have been identified, but exactly how these proteins act on lipid bilayers to generate and maintain the unique ER membrane structures is still not well understood.

Sey1p from the yeast *Saccharomyces cerevisiae* is a dynamin-related GTPase that binds to ER membranes through its two transmembrane domains at the C-terminus (Fig. 1a) and is thought to be a functional ortholog of the atlastin family of GTPases in animals, which are reportedly required for homotypic ER fusion and thereby generate the tubular ER network^6^,^7^,^10^,^11^. Recent reconstitution studies have established that both of these ER-bound dynamin-related GTPases, atlastins and Sey1p in yeast, have the capacity to induce lipid bilayer fusion directly^10^,^11^,^12^,^13^,^14^, even though atlastins and Sey1p share relatively low sequence homology (12% overall identity) and a substantial difference in the length of the helical middle regions between the GTPase and transmembrane domains^11^ (Fig. 1a). To obtain deeper mechanistic insights into this GTPase-dependent ER fusion process, particularly focusing on the roles of lipid molecules, here we developed *in vitro* membrane docking and lipid mixing assays using proteoliposomes reconstituted with purified recombinant Sey1p (Fig. 1b,c) and synthetic liposomes bearing the ER-mimicking lipid set^15^,^16^,^17^, containing phosphatidylcholine (PC), phosphatidylethanolamine (PE), phosphatidylinositol (PI), phosphatidylserine (PS), phosphatidic acid (PA), ergosterol (ERG), cardiolipin (CL), and diacylglycerol (DAG) (Table 1 and Fig. 2). The present study using a chemically defined system of reconstituted proteoliposomes revealed that complex but physiological lipid composition is strictly required for homotypic ER membrane fusion mediated by the dynamin-related GTPase Sey1p in yeast.

## Results and Discussion

### *In vitro* reconstitution of proteoliposomal membrane docking and lipid mixing mediated by Sey1p-GTP

**Figure 1:**
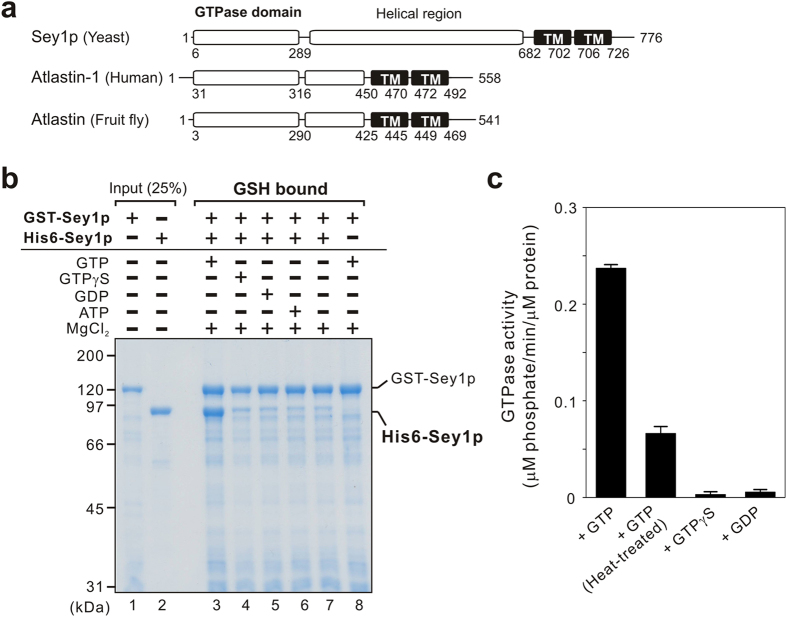
The dynamin-related GTPase Sey1p specifically forms a homo-oligomer in solution in a GTP-dependent manner. (**a**) Schematic representation of the dynamin-related GTPase Sey1p in yeast and its functional orthologues in humans (Atlastin-1) and the fruit fly (Atlastin), showing the amino acid residues and domains (GTPase and transmembrane domains). Helical bundles of the dynamin-related GTPases are present in the middle regions between GTPase domains and transmembrane domains. (**b**) Purified recombinant Sey1p specifically assembles into a homo-oligomer in the presence of GTP. Purified GST-Sey1p (lane 1; final concentration, 4 μM) and His6-Sey1p (lane 2; final concentration, 4 μM) were mixed in RB150 (20 mM Hepes-NaOH, pH 7.4, 10% glycerol, 150 mM NaCl) containing 2 mM MgCl_2_ and 0.4% Triton X-100, in the presence of 1 mM GTP (lanes 3 and 8), GTPγS (lane 4), GDP (lane 5), or ATP (lane 6) or in the absence of any nucleotides (lane 7). GST-Sey1p was then isolated using glutathione-Sepharose beads and subjected to SDS-PAGE and Coomassie Blue staining to analyze co-isolated His6-Sey1p proteins bound to GST-Sey1p. (**c**) Intrinsic GTP hydrolysis activity of purified recombinant Sey1p. The GTPase activity of Sey1p (final concentration, 16 μM) was assayed in RB500 (20 mM Hepes-NaOH, pH 7.4, 10% glycerol, 500 mM NaCl) containing 0.4% Triton X-100, 2 mM MgCl_2_, and 1 mM GTP, using a Malachite Green-based reagent. As the control, heat-denatured Sey1p for proteins and GTPγS or GDP for nucleotides were added where indicated.

We purified GST-tagged and His6-tagged full-length Sey1p from *E. coli* expression systems (Fig. 1b, lanes 1 and 2) and then initially employed GST pull-down assays and GTPase activity assays using purified Sey1p (Fig. 1b, lanes 3–8; Fig. 1c) to establish that the recombinant Sey1p proteins in our current preparations were well folded and functionally active. The purified Sey1p proteins in the present study exclusively assembled into a homo-oligomeric complex in the presence of GTP, as His6-Sey1p was co-isolated specifically with GST-Sey1p when GTP was added to the GST pull-down assays (Fig. 1b, lane 3), and it retained intrinsic GTPase activity (Fig. 1c). It is noteworthy that we observed very stringent GTP requirements for Sey1p oligomerization even in a *cis* configuration, as both GTPγS and GDP had little potency to support the stable interactions between full-length Sey1p proteins in detergent solution (Fig. 1b, lanes 4 and 5).

**Figure 2:**
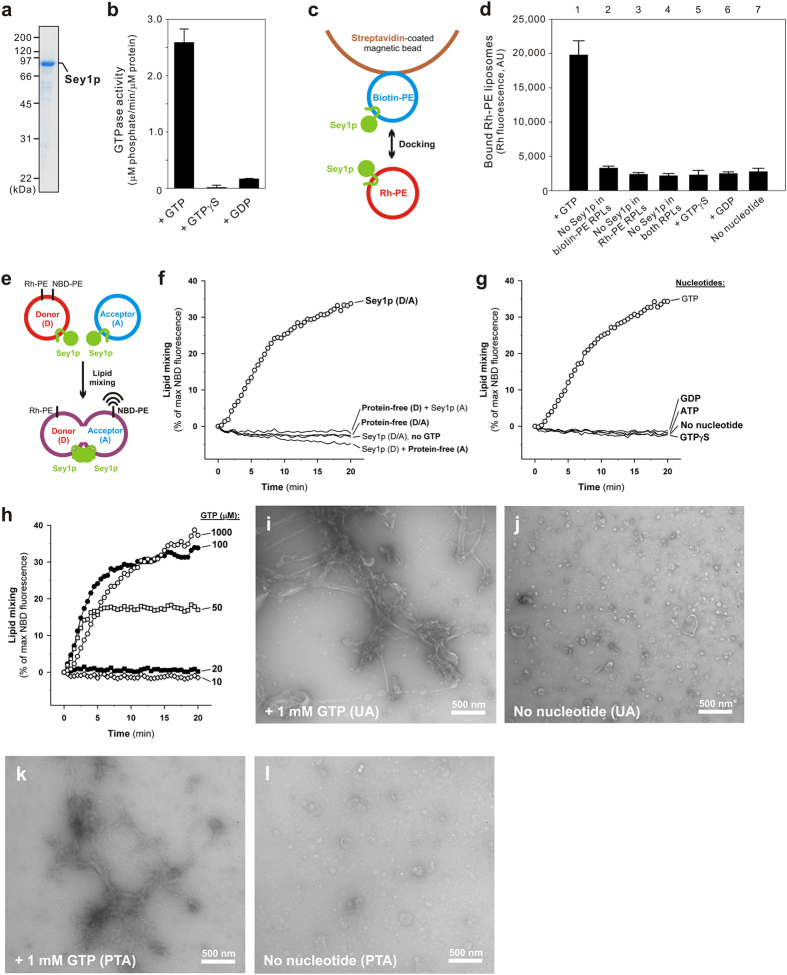
Reconstitution of Sey1p-mediated proteoliposomal membrane docking and lipid mixing. (**a**) Coomassie Blue-stained gel showing proteoliposomes bearing Sey1p and the ER-mimicking lipids. (**b**) The Sey1p proteins reconstituted into proteoliposomes retain GTPase activity. GTPase activity of Sey1p proteoliposomes was assayed, as in Fig. 1c. The concentrations of Sey1p were estimated using a protein-to-lipid ratio of 1/500 (mol/mol). (**c**) Schematic representation of the membrane docking assay using streptavidin-coated beads and Sey1p proteoliposomes bearing either biotin-labeled or Rh-labeled PE. (**d**) Sey1p proteins on two opposing membranes mediate GTP-dependent membrane docking. The biotin-labeled Sey1p proteoliposomes, the Rh-labeled Sey1p proteoliposomes, and streptavidin-coated beads were mixed and incubated in RB500 containing 2 mM MgCl_2_ and 1 mM GTP (lane 1). The Rh-labeled liposomes that bound to the biotin liposomes were analyzed by measuring the fluorescence of Rh. As a control, protein-free liposomes bearing biotin-PE (lanes 2 and 4) or Rh-PE (lanes 3 and 4), GTPγS (lane 5), and GDP (lane 6) were added to the reactions. (**e**) Schematic representation of the lipid mixing assay used to monitor dequenching of the NBD fluorescence of Sey1p proteoliposomes. (**f**) Sey1p proteins on two opposing membranes induce efficient lipid mixing in the presence of GTP. Lipid mixing was assayed in RB150 containing 1 mM GTP and 2 mM MgCl_2_, with the Rh/NBD-labeled donor Sy1p proteoliposomes and the non-labeled acceptor Sey1p proteoliposomes. (**g**) Sey1p-mediated lipid mixing requires not only GTP binding but also GTP hydrolysis. Lipid mixing was assayed as in (**f**), in the presence of 1 mM GTP, GDP, GTPγS, or ATP. (**h**) Sey1p-mediated lipid mixing strictly depends on the concentration of GTP. Lipid mixing was assayed as in (**f**), in the presence of various concentrations of GTP. (**i–l**) Negative staining electron microscopy analysis of the lipid mixing reactions of reconstituted Sey1p proteoliposomes. The donor and acceptor proteoliposomes bearing Sey1p were mixed, incubated in the presence (**i,k**) or absence (**J,l**) of 1 mM GTP, and negatively stained with 1% uranyl acetate (UA) (**i,j**) or phosphotungstic acid (PTA) (**k,l**). Scale bars: 500 nm.

Sey1p-bearing proteoliposomes were reconstituted with the untagged form of Sey1p, which had been digested by human rhinovirus 3C protease to cleave the N-terminal polyhistidine tag, and with the preformed protein-free liposomes bearing the ER-mimicking lipids (Table 1), using a detergent-assisted insertion method^6^,^18^. Active full-length Sey1p with its two C-terminal transmembrane domains was successfully incorporated into liposomal membranes (Fig. 2a) and exhibited intrinsic GTP-hydrolyzing activity on the reconstituted proteoliposomes (Fig. 2b). As we recently developed an *in vitro* assay to monitor membrane tethering among human Rab GTPase-anchored liposomes^19^, we extended and modified this assay to allow evaluation of Sey1p-mediated membrane docking (Fig. 2c). After incubating the reaction mixtures containing the biotin-labeled and rhodamine (Rh)-labeled liposomes bearing Sey1p with streptavidin-coated beads (Fig. 2c), Rh fluorescence of the co-isolated Rh/Sey1p and biotin/Sey1p liposomes was measured to quantify the physical interactions between these Sey1p liposomes (Fig. 2d). This current membrane docking assay detected stable and efficient Sey1p-dependent proteoliposomal docking (Fig. 2d, lane 1 vs. lane 4) and clearly demonstrated a strict requirement for *trans*-Sey1p assembly on opposing membranes (Fig. 2d, lane 1 vs. 2–3) and GTP binding/hydrolysis (Fig. 2d, lane 1 vs. 5–7) on membrane docking, consistent with earlier reconstitution studies on atlastins^20^,^21^.

Next, we examined the fusogenic capacity of Sey1p to induce lipid bilayer rearrangement of proteoliposomes by employing a well-established fluorescence lipid mixing assay^6^,^22^,^23^,^24^,^25^ (Fig. 2e). Sey1p indeed exhibited intrinsic fusogenic potency, directly mediating rapid lipid mixing of the reconstituted proteoliposomes (Fig. 2f–h). This Sey1p-dependent lipid mixing strictly requires GTP binding/hydrolysis and the presence of Sey1p proteins on both opposing membranes destined for fusion (Fig. 2f,g), as expected from the results from our current membrane docking assays (Fig. 2d). Intriguingly, even relatively low GTP concentrations, e.g., 50–100 μM, were sufficient for Sey1p to initiate efficient lipid mixing, whereas the Sey1p-dependent lipid mixing at 50 μM GTP was drastically diminished after 5 min of incubation; moreover, Sey1p was no longer fusogenic at the onset of the reactions at or below 20 μM GTP (Fig. 2h). These findings reflect that Sey1p-dependent fusogenicity is highly sensitive to the concentration of GTP and requires continuous GTP hydrolysis, as reported previously in atlastin GTPase reconstitution studies^20^,^21^. In addition to the conventional fluorescence lipid mixing assay, for further validation of the fusogenic potency of Sey1p, the lipid mixing reactions of Sey1p-bearing proteoliposomes were analyzed by electron microscopy using a negative staining method (Fig. 2i–l). We observed that Sey1p indeed caused massive rearrangement of proteoliposomal lipid bilayers when 1 mM GTP was added, thereby generating characteristic reticular and/or tubular membrane structures with some three-way junctions (Fig. 2i–l). The data obtained from these two distinct and independent experimental approaches, a lipid mixing assay and negative-staining electron microscopy, showed that the Sey1p GTPase has intrinsic fusogenic capacity to trigger GTP-dependent membrane fusion directly by itself. It is also noteworthy that the present observations confirmed that the fluorescence lipid mixing assay (Fig. 2e) is a reliable method for assessing the fusogenicity of reconstituted proteoliposomes, at least in the case of Sey1p-dependent fusion reactions, despite the caveats of this assay reported in recent reconstitution studies of SNARE proteins^26^,^27^.

### Physiological lipid composition is required for Sey1p-mediated membrane fusion

**Figure 3:**
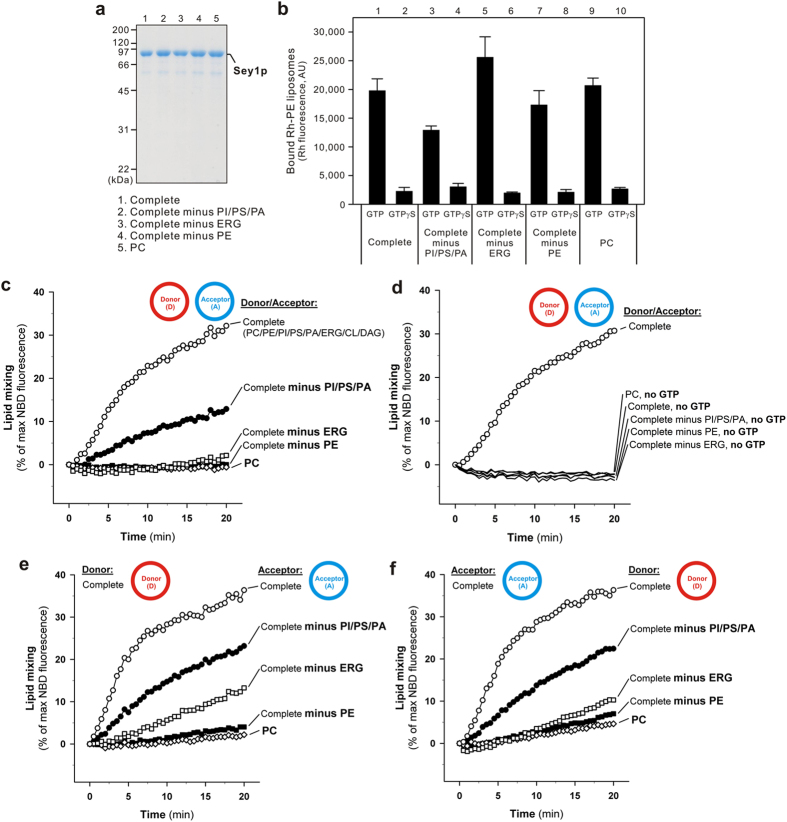
Requirement of a physiological complex lipid composition for Sey1p-mediated membrane docking and lipid mixing. (**a**) Coomassie Blue-stained gel showing the reconstituted Sey1p proteoliposomes used in (**b–f**), which harbored various sets of lipids (Table 1). (b) Lipid composition is not critical for Sey1p-mediated membrane docking. Sey1p-dependent proteoliposomal docking was assayed as in Fig. 2d, using the Sey1p proteoliposomes bearing various sets of lipids (Table 1). Data on the Sey1p liposomes with the complete ER-mimicking lipid set (lanes 1 and 2) are the same as the data shown in Fig. 2d. (**c,d**) A complex but physiological lipid composition is vital for Sey1p-mediated proteoliposomal lipid mixing. Lipid mixing was assayed as in Fig. 2f-h, using the Sey1p liposomes bearing various sets of lipids (Table 1), in the presence (**c**) or absence (**d**) of 1 mM GTP. (**e,f**) Specific lipid molecules (PI/PS/PA, ERG, and PE) are required on both opposing membranes for efficient lipid mixing mediated by Sey1p. Lipid mixing was assayed as in (**c**), except using the Sey1p liposomes bearing the complete lipid set for either the donor (**e**) or the acceptor (**f**) liposomes.

The importance of the complex physiological lipid composition of eukaryotic cells in membrane docking and fusion processes has been investigated extensively using chemically pure proteoliposomal systems containing purified SNARE proteins, SNARE-binding chaperones, Rab GTPases, and defined lipid mixes^28^. These reconstitution studies on SNARE-bearing proteoliposomes reflect that specific lipid species, including phosphoinositides, acidic lipids, and non-bilayer lipids, directly facilitate SNARE-mediated membrane fusion^23^,^29^,^30^,^31^,^32^,^33^,^34^,^35^. Using current newly developed assays to analyze Sey1p-dependent proteoliposomal membrane docking (Fig. 2c,d) and lipid mixing (Fig. 2e–h), we next explored the essential role of lipids in homotypic ER membrane fusion mediated by the Sey1p GTPase and GTP (Fig. 3, Table 1). In addition to the ER-mimicking lipids depicted in Fig. 2, we further applied four distinct lipid subsets from the complete ER-mimicking lipid set to reconstitute Sey1p-bearing liposomes: PC alone and three sets of lipid mixtures lacking three major acidic phospholipids (PI, PS, and PA), ERG, or PE (Table 1). In all cases, these lipid compositions did not significantly affect the amount of Sey1p proteins reconstituted into proteoliposomes (Fig. 3a). Exploiting the streptavidin bead-based liposome docking assay (Fig. 2c), all of the lipid compositions evaluated, even PC alone, retained comparable capacities for supporting GTP-dependent membrane docking of Sey1p-bearing liposomes (Fig. 3b, lanes 1, 3, 5, 7, and 9). These findings strongly suggest that membrane docking mediated by the Sey1p GTPase is completely independent of the lipid composition of lipid bilayers, and thus protein-protein interactions involved in Sey1p homo-oligomerization can drive stable proteoliposomal membrane docking. Nevertheless, as shown below, lipid composition is crucial for Sey1p-mediated lipid mixing (Fig. 3c–f).

To investigate directly whether specific lipid species are indispensable for the fusogenicity of Sey1p-mediated docked membranes, we next employed lipid mixing assays for Sey1p proteoliposomes reconstituted with five of the distinct lipid compositions (Fig. 3c–f, Table 1), as evaluated in the membrane docking assays (Fig. 3b). Strikingly, we revealed the critical importance of a physiological and complex lipid composition for Sey1p-dependent membrane fusion (Fig. 3c,d): Sey1p proteoliposomes containing PC alone were completely incapable of initiating lipid mixing (Fig. 3c, open diamonds). The omission of three acidic lipids, PI, PS, and PA, significantly reduced the initial rate of lipid mixing (Fig. 3c, filled circles), and more remarkably, the single omission of either ERG (Fig. 3c, open squares) or PE (Fig. 3c, filled squares) entirely abolished the fusogenic potency of Sey1p. Furthermore, even when the mixed combinations of the complete ER-mimicking liposomes for donor (Fig. 3e) or acceptor (Fig. 3f) and their partner liposomes lacking specific lipids (PI/PS/PA, ERG, or PE) were assayed, we still observed very strict lipid requirements for the fusogenic potency of Sey1p (Fig. 3e,f), indicating that efficient Sey1p-dependent membrane fusion requires a certain physiological lipid composition for both of the opposing lipid bilayers that are destined to fuse. Altogether, we established that, in addition to the dynamin-related Sey1p GTPase and GTP, these specific lipids (ERG, PE, and the three major acidic lipids PI, PS, PA) are also key components for homotypic ER membrane fusion in yeast, which function in lipid bilayer rearrangement after Sey1p-GTP-mediated membrane docking (Figs 2 and 3).

How can these lipid molecules specifically support fusion? Earlier reconstitution studies of the functional orthologue atlastin GTPase revealed that the C-terminus of atlastin forms a conserved amphipathic helix that stimulates fusion by directly associating with and perturbing the lipid bilayer^12^,^36^. This leads us to postulate that the acidic lipids interact with positively charged residues in the C-terminus of Sey1p, thereby partially supporting its amphipathic helix formation and specific fusion functions. The important role of PE in membrane fusion has been directly demonstrated by proteoliposomal studies on yeast vacuolar SNAREs, in which the removal of PE from the vacuole lipid mixture largely diminished the rate of SNARE-dependent lipid mixing or membrane fusion^31^,^35^. Since PE is a cone-shaped, non-bilayer lipid with a small polar headgroup^37^, we assume that it supports fusion simply by inducing curvature stress and a non-bilayer structure in the membrane, in cases of both SNARE- and Sey1p-dependent fusion. It is noteworthy that the single omission of ERG from the ER-mimicking lipids abolished Sey1p-mediated lipid mixing completely (Fig. 3c, open squares), whereas ERG contributed little to the stable membrane docking mediated by Sey1p and GTP (Fig. 3b, lanes 5–6). Sterols, including ERG in yeast and cholesterol in mammals, stabilize membrane microdomains^37^ and indeed contribute to the enrichment of fusion factors in the microdomains^38^,^39^. Furthermore, the requirement of cholesterol-rich lipid domains for membrane fusion was recently reported for a chemically defined system involving the human immunodeficiency virus fusion peptide^40^. Thus, we hypothesize that ERG may cooperate with Sey1p in establishing fusion-competent membrane microdomains, thereby defining the sites of three-way junctions in ER membrane structures.

## Methods

### Protein expression and purification

The coding sequence of the full-length yeast Sey1p protein was amplified by polymerase chain reaction (PCR) from the genomic DNA of the yeast *S. cerevisiae* BY4741 strain, using two DNA primers (5′-GAC GAC GAC AAG ATG GGT GCA CTT GAA GTC CTC TTT CAG GGA CCC GGT ATG GCT GAT AGA CCT GC-3′; 5′-GAG GAG AAG CCC GGT TCA TTT TTC TTT TTG CTC-3′), and then cloned into the pET-30 Ek/LIC or pET-41 Ek/LIC vector (Novagen) expressing a His6- or GST-His6-tagged protein, respectively. The amplified PCR fragment contained a human rhinovirus 3C protease site (Leu-Glu-Val-Leu-Phe-Gln-Gly-Pro) upstream of the initial ATG codon, yielding the full-length, untagged form of Sey1p with only three extra N-terminal residues (Gly-Pro-Gly) after 3C protease cleavage. Recombinant Sey1p proteins were produced in the *Escherichia coli* Rosetta 2(DE3)pLysS strain (Novagen), cultured in Terrific Broth medium (1 L each) containing kanamycin and chloramphenicol, by induction with 1 mM iso-propyl 1-thio-β-D-galactopyranoside at 37 °C for 2 h for GST-His6-3C-Sey1p and at 16 °C for 20 h for His6-3C-Sey1p. *E. coli* cells were harvested and resuspended in 40 ml buffer A (20 mM sodium phosphate, pH 7.0, 500 mM NaCl, 10% glycerol, 1 mM EDTA) containing 2% Triton X-100, 1 mM dithiothreitol, 1 mM phenylmethylsulfonyl fluoride, and 1.0 μg/ml pepstatin A. Cell suspensions were incubated at 4 °C for 30 min with gentle shaking, lysed by sonication, and centrifuged at 50,000 rpm for 1 h at 4 °C using a 70 Ti rotor (Beckman Coulter). The resulting supernatants were mixed with a 50% slurry of COSMOGEL His-Accept beads (Nacalai Tesque) in buffer A containing 0.4% Triton X-100 (10 ml each) and were incubated at 4 °C for 2 h with gentle agitation. After washing the beads in buffer A containing 0.4% Triton X-100 and 20 mM imidazole, His6-3C-Sey1p or GST-His6-3C-Sey1p was eluted with buffer A containing 0.4% Triton X-100 and 500 mM imidazole, followed by dialyzing the eluted fractions against RB150 (20 mM Hepes-NaOH, pH 7.4, 150 mM NaCl, 10% glycerol) containing 0.4% Triton X-100 and 1 mM EDTA.

### GST pull-down assay

Purified recombinant GST-His6-3C-Sey1p and His6-3C-Sey1p proteins were each mixed at 4 μM in 500 μl RB150 containing 0.4% Triton X-100, MgCl_2_ (2 mM), and either GTP (1 mM), GTPγS (1 mM), GDP (1 mM), or ATP (1 mM) where indicated, incubated at 4 °C for 1 h with gentle agitation, mixed with glutathione-Sepharose 4B beads (200 μl, 50% slurry; GE Healthcare) equilibrated in RB150 containing 0.4% Triton X-100 and 2 mM MgCl_2_, and further incubated at 4 °C for 1 h. The glutathione-Sepharose beads were isolated by centrifugation (2 min, 15,300 *g*, 4 °C) and washed four times in 400 μl RB150 containing 0.4% Triton X-100 and 2 mM MgCl_2_. GST-His6-3C-Sey1p and His6-3C-Sey1p proteins bound to the beads were eluted at 100 °C for 5 min with 2% SDS, and then subjected to SDS-PAGE and Coomassie Blue staining.

### GTPase activity assay

The GTP hydrolysis activity of purified recombinant Sey1p was assayed by quantitating the released free phosphate molecules, using the Malachite Green-based reagent Biomol Green (Enzo Life Sciences). The untagged form of Sey1p (final concentration, 16 μM), which had been digested by human rhinovirus 3C protease (Novagen) at 4 °C for 16 h in RB150 containing 1 mM EDTA and 0.4% Triton X-100, was incubated at 30 °C for 30 min in 100 μl RB500 (20 mM Hepes-NaOH, pH 7.4, 500 mM NaCl, 10% glycerol) containing 0.4% Triton X-100, 2 mM MgCl_2_, and either GTP (1 mM), GTPγS (1 mM), or GDP (1 mM) where indicated. The reaction mixtures (100 μl each) were then diluted three-fold with RB500 containing 0.4% Triton X-100, supplemented with 600 μl Biomol Green reagent, incubated at 30 °C for 15 min, and measured for absorbance at 620 nm using the DU720 spectrophotometer (Beckman Coulter). The Sey1p protein denatured by treatment at 100 °C for 5 min was also assayed using the same protocol. To assess the GTPase activity of the reconstituted proteoliposomes bearing untagged Sey1p proteins, Sey1p liposomes (final lipid concentration, 700 μM) were incubated at 30 °C for 30 min in 50 μl RB150 containing 2 mM MgCl_2_ and either GTP (1 mM), GTPγS (1 mM), or GDP (1 mM) where indicated, diluted six-fold with RB150, and then subjected to the assays described above using purified Sey1p in detergent solution. All of the data were corrected by subtracting the absorbance values obtained from the control reactions without any guanine nucleotides. Means and standard deviations of the corrected values (ΔA620) were determined from three independent experiments.

### Preparation of reconstituted Sey1p-bearing proteoliposomes

The reconstitution of proteoliposomes bearing the dynamin-related GTPase Sey1p was employed as described previously for the atlastin-family GTPases, with some modifications^6^,^36^. The non-fluorescent lipids were from Avanti Polar Lipids, except for ergosterol, which was obtained from Sigma. Fluorescent lipids, N-(7-nitro-2,1,3-benzoxadiazole-4-yl)-PE (NBD-PE), N-(lissamine rhodamine B sulfonyl)-PE (Rh-PE), and dansyl-PE, were obtained from Molecular Probes. The ER-mimicking lipid mixes used for Sey1p liposomes contained 1-palmitoyl-2-oleoyl-PC (POPC) (44%, 46%, or 43% (mol/mol) for donor, acceptor, or biotin-labeled proteoliposomes, respectively), POPE (20%), soy PI (10%), POPS (8%), POPA (3%), ERG (10%), bovine CL (1.0%), DAG (1.0%), and fluorescent lipids (1.5% each of NBD-PE/Rh-PE or 1.0% of dansyl-PE for donor or acceptor and biotin-labeled liposomes, respectively). Although we used palmitoyl-oleoyl lipids for most of the lipid species in the preparation of reconstituted Sey1p proteoliposomes, it should be noted that yeast subcellular membranes contain substantial amounts of diunsaturated lipid species^17^. Dried lipid films harboring these ER-mimic lipid compositions were hydrated in RB150 with 1 mM EDTA to a total lipid concentration of 8 mM, incubated at 37 °C for 1 h with shaking, subjected to six freeze-thaw cycles in liquid N_2_ and a water bath at 37 °C, and extruded 11 times through polycarbonate filters (pore size, 100 nm) in a mini-extruder (Avanti Polar Lipids) at 40 °C. To reconstitute Sey1p-bearing proteoliposomes, protein-free liposomes prepared by extrusion as described above (final lipid concentration, 3.8 mM) were mixed with the untagged full-length form of Sey1p (final concentration, 7.6 μM), which had been digested by human rhinovirus 3C protease (Novagen) at 4 °C for 16 h, in 400 μl RB150 containing 1 mM EDTA and 0.1% (1.7 mM) Triton X-100. Under these experimental conditions, the effective detergent-to-lipid ratio (*R*_eff_) was calculated to be 0.39 (mol/mol), using the equation *R*_eff_ = (*D*_total_ − *D*_water_)/[lipid], in which *D*_total_ is the total detergent concentration, *D*_water_ is the aqueous monomeric detergent concentration (0.18 mM for Triton X-100), and [lipid] is the lipid concentration^18^. The current *R*_eff_ value used was less than the detergent-to-lipid ratio of detergent-saturated liposomes (*R*_sat_) for Triton X-100 of 0.64 (mol/mol)^18^. The detergent-liposome-protein mixed solutions (400 μl each) were incubated at 4 °C for 1 h with gentle agitation, supplemented with Bio-beads SM-2 adsorbent beads (0.05 g for each; Bio-Rad) to remove the detergent, and incubated (4 °C, 2 h, gentle agitation), followed by a second round of incubation with fresh Bio-beads (4 °C, 16 h, gentle agitation). Unincorporated insoluble protein aggregates were separated from proteoliposomes by centrifugation, as described previously^6^. The Sey1p-bearing proteoliposomes formed were harvested, diluted with RB150 containing 1 mM EDTA (2 mM total lipids), and stored at −80 °C. Lipid concentrations of the Sey1p proteoliposomes were determined according to the fluorescence of NBD-PE or dansyl-PE, as described previously^23^.

### Membrane docking assay

Membrane docking assays were performed using streptavidin-coated magnetic beads, as described previously^19^, with modifications. The biotin-labeled Sey1p-bearing proteoliposomes (final lipid concentration, 500 μM) were mixed with streptavidin-coated magnetic beads (Dynabeads M-280 Streptavidin; Invitrogen) and the Rh-labeled Sey1p proteoliposomes (final lipid concentration, 500 μM) in 100 μl RB500 containing 2 mM MgCl_2_ and 1 mM GTP, GTPγS, or GDP, where indicated, and then incubated at 30 °C for 30 min with gentle agitation. The proteoliposome-bound beads were isolated using a DynaMag magnet (Invitrogen) and resuspended in RB500 containing 0.4% Triton X-100 to solubilize the liposomes bound to the beads. To quantify the Rh-labeled liposomes co-isolated with the biotin-labeled liposomes, Rh fluorescence (λ excitation = 560 nm, λ emission = 580 nm, emission cut-off = 570 nm) in the supernatants obtained was measured using the SpectraMAX Gemini XPS plate reader (Molecular Devices). Means and standard deviations of the Rh fluorescence signals were obtained from three independent experiments.

### Lipid mixing assay

Lipid mixing assays were performed using NBD-labeled Sey1p-bearing donor liposomes and unlabeled Sey1p-bearing acceptor liposomes, as described previously^22^,^23^,^24^,^25^, with modifications. Donor Sey1p liposomes (final lipid concentration, 200 μM) and acceptor Sey1p liposomes (final lipid concentration, 500 μM) were mixed in RB150 in a black 384-well plate (no. 3676; Corning) and preincubated at 30 °C for 10 min in the SpectraMAX Gemini XPS plate reader (Molecular Devices). After preincubation, GTP (0.01–1 mM), GTPγS (1 mM), GDP (1 mM), ATP (1 mM), and MgCl_2_ (2 mM) were added to the reactions where indicated, followed by further incubation at 30 °C for 30 min. NBD fluorescence (λ excitation = 460 nm, λ emission = 538 nm, emission cut-off = 515 nm) was measured at 30-s intervals, with 30 reads per well on the ‘middle’ PMT setting (arbitrary units), to monitor lipid mixing. β-OG (final concentration, 100 mM) was added after incubation for 30 min to obtain fully dequenched maximal NBD fluorescence. The ratios of NBD fluorescence (%) were calculated as described previously^23^. All lipid mixing data shown in Figs 2 and 3 are from one experiment and are typical of those from more than three independent experiments.

### Negative staining electron microscopy

The donor (final lipid concentration, 200 μM) and acceptor (final lipid concentration, 500 μM) Sey1p proteoliposomes bearing the complete set of ER-mimicking lipids (see Table 1) were mixed in RB150, preincubated at 30 °C for 10 min, supplemented with GTP (final concentration, 1 mM) and MgCl_2_ (final concentration, 2 mM), and further incubated at 30 °C for 30 min. The incubated proteoliposomal reactions were diluted five-fold with RB150, and a drop of the diluted reactions (5 μl each) was applied to a Formvar-coated copper grid. These proteoliposome samples were negatively stained with 1% uranyl acetate or phosphotungstic acid, followed by the removal of excess staining solution using filter paper and air-drying at room temperature. Images of the negatively stained samples were obtained using the JEOL JEM-1011 transmission electron microscope.

## Additional Information

**How to cite this article**: Sugiura, S. and Mima, J. Physiological lipid composition is vital for homotypic ER membrane fusion mediated by the dynamin-related GTPase Sey1p. *Sci. Rep.* **6**, 20407; doi: 10.1038/srep20407 (2016).

## Figures and Tables

**Table 1:** Lipid compositions of the reconstituted Sey1p-bearing proteoliposomes used in this study.

Lipid compositions	Lipids^1^							
	POPC^2^	POPE	SoyPI	POPS	POPA	ERG	CL	DAG
% (mol/mol)								
Complete	44/46/43	20	10	8	3	10	1	1
Complete minus PI/PS/PA	65/67/64	20	—	—	—	10	1	1
Complete minus ERG	54/56/53	20	10	8	3	—	1	1
Complete minus PE	64/66/63	—	10	8	3	10	1	1
PC	97/99/96	—	—	—	—	—	—	—

^1^Donor and acceptor liposomes contain the fluorescent lipids, NBD-PE/Rh-PE (1.5% each) and dansyl-PE (1%), respectively. Biotin-labeled liposomes for membrane docking assays contain dansyl-PE (1%) and biotin-PE (3%).

^2^The percentages of POPC for each lipid composition are shown as % for donor liposomes/% for acceptor liposomes/% for biotin-labeled liposomes.
